# Natura Non Facit Saltum

**Published:** 1891-05-09

**Authors:** 


### The Doctor's Armchair.

"NATURA NON FACIT SALTUM."

Count Moltke has for twenty years been one of the world's most interesting and striking figures. The man, of whatever nationality, who has read the story of his long life, and of his fitting death, without sympathy and without veneration is not to be envied. To all people who think soberly of the times in which we live, and who wish to judge justly, Count Moltke is a sign, a finger-post, an object on the landscape of humanity, full of significance and of instruction.

Medicine, by which we here mean sick people, their doctors, and their nurses, has something to learn from Count Moltke. The words placed at the head of this article may be freely paraphrased thus: "Nature does not advance by flying leaps, like a kangaroo." This is a dictum of Count Moltke's at ninety years of age. A German author living in London sent an essay on a social question to the Count some months ago. The words we have paraphrased constituted a part of the answer received. "Nature does not advance by leaps," says the Count, "neither does civilisation." To which we may add—neither does medicine.

Undisciplined minds love wonders and are easily persuaded to rush after them. A Sequah can command five thousand hearers on a village green. But we do not expect disciplined minds, especially minds that have been scientifically trained, to rush after wonders and portents. Experience and science teach us that those things which have happened are the very things which are most likely to happen again, and that anything which appears unusual is to be regarded first of all with suspicion. But of all the seekers after wonders and miracles of the present period, a certain class of medical men are the chief. The paths of experience and sound observation have no longer any charm for them: they insist that nature shall conjure for their fame and profit. In proof of this we have but to name the name of Koch. Following the impulsive, the undisciplined, the unscientific example of the younger medical men of the time, the mass of the public, because they are ignorant and know no better, are constantly on the tip-toe of expectation for new medical wonders.

If experience justified us in looking for discoveries of a practically miraculous kind, undisciplined doctors and an ignorant public would have a reasonable excuse to plead. But the world has been in existence a fairly long time; and there have been in many ages and countries men of such intellectual capacity and faculty for observation as cannot be surpassed and perhaps hardly equalled, even in our own pre-eminently scientific period. The average youthful medical scientist is to be profoundly pitied in that he usually knows nothing at all but the opinions of his own particular teacher, and the practice of his own little laboratory. He works for the most part in a scientific tomb. What has been done outside the walls of his tomb he has never learnt, and, unfortunately, he does not care to learn. The only sound from the great external world to which he pays any attention, if he be an Englishman, is some faint and far-off echo from modern German laboratories. It is very doubtful, however, whether his German confrère pays him the same compliment of respect for his work. But the scientific Briton and the German of the present time are both alike in this, that they are almost destitute of the power of generalisation. In one thing they are absolutely agreed; and that is in a conspiracy of contempt, both for the large facts of nature and for the great scientific generalisations of past thinkers and workers.

*Natura non facit saltum.* Whoever has an ear for truth catches its tones here. Moltke, rare old man, spoke what he knew, and testified what he had seen. Is nature our servant, or is she our mistress; is she our pupil, or is she our teacher? If she is ever our servant, it is only when, like a mighty queen, she shows her royal character by making herself serviceable to the general good. Nature is our mistress; our teacher. When we are docile she teaches us truth, and we learn it and know it; and then, too, she permits us to make use of her in a hundred ways for our profit. But whoever thinks that he is able to set up a standard of successful revolt, and to substitute a human purpose and will for the ordered and inevitable progress of nature is like a kitten in a thirty feet deep well: He may flounder and mew for a few brief minutes, but he will certainly end by being drowned.

Medical men often fail in science because their eye is not single. "If thine eye be single," said the most wonderful of all generalisers, "thy whole body shall be full of light." The aim of the medical man is too frequently not science because science is serviceable to man, but science because it will bring him reputation and fortune. He does not understand that it is as impossible to serve science and mammon as it is to serve God and mammon. This double motive produces two injurious results: it distracts attention from what should be the main object of scientific work, and it makes the worker impatient for the lowest form of reward. If Koch had had the patience of the true man of science he would not now have been an object of pity to thousands of insignificant persons who were eager to crown him a divinity less than six months ago.

He who cannot wait is not fit for any of the world's great work. Moltke was sixty-five before Europe had any knowledge of his great powers and personality. Men may make discoveries in science and medicine, just as they may win battles, in comparative youth: but they cannot discern the true proportions of their own work, and its relative value in the world, until the eyes of their experience have surveyed wide fields of knowledge, and until their judgment has been matured by long training and practice. How few medical men fulfil these conditions! Most of them are craftsmen, and they do not know it. They delude themselves with the idea that they are men of science. It is no dishonour to them to be craftsmen if they are skilful at their craft: on the contrary, it is honourable. But the mere craftsman, be he medical or otherwise, has no more right to the robe and crown of science than the ass had to the skin of the lion.

It cannot be affirmed that youthful medical scientists have a monopoly of undisciplined impatience. The rapid increase of population and the quick growth of wealth are not conducive to mental steadiness and sobriety. The eager man rushes forward with confident recklessness, and tilts his sober neighbour into the gutter. The reckless runner thinks the sober man a fool. The sober man knows that the reckless runner is a fool. The great world will generally put its money on the reckless runner, at any rate for a time, all the same; and it stands to lose a good deal by the process. But, in spite of innumerable temptations "to rush his work and his career," the man of enduring sense and strength believes in nature, follows her methods, is guided by the large generalisations which she objectively shows him; and at length duly emerges, almost alone, at the mountain top of knowledge, judgment, and practical capacity. "He that believeth," says the venerable book, "shall not make haste;" to which we may add—He that understandeth shall not make haste. *Natura non facit saltum.*

